# Effect of gum-chewing exercise on maintaining and improving oral function in older adults: A pilot randomized controlled trial

**DOI:** 10.1016/j.jds.2023.06.029

**Published:** 2023-07-09

**Authors:** Kenta Kashiwazaki, Yuriko Komagamine, Yoko Uehara, Mao Yamamoto, Hiroto Nakai, Ngoc Huyen Trang Bui, Hengyi Liu, Sahaprom Namano, Watcharapong Tonprasong, Manabu Kanazawa, Shunsuke Minakuchi

**Affiliations:** a Gerodontology and Oral Rehabilitation, Graduate School of Medical and Dental Sciences, Tokyo Medical and Dental University, Tokyo, Japan; b Digital Dentistry, Graduate School of Medical and Dental Sciences, Tokyo Medical and Dental University, Tokyo, Japan

**Keywords:** Chewing gum, Bite force, Aged, Randomized controlled trial

## Abstract

**Background/purpose:**

Gum chewing has been found to improve oral function. Nevertheless, few randomized controlled trials have investigated the effects of gum-chewing exercises on oral function in older adults. This study aimed to examine the effect of gum-chewing exercises on oral function in older adults.

**Materials and methods:**

This was a single-blind, randomized controlled trial, conducted from November 2021 to January 2022. A total of 130 participants were divided randomly into the intervention and control groups. The intervention group was told to chew experimental gums for one month, while the control group was instructed to chew experimental tablets for one month. Maximum bite force, occlusal contact areas, oral dryness, tongue pressure, tongue and lip functions (number of times each of the following syllables is pronounced per second:/pa/,/ta/, and/ka/), masticatory function, subjective masticatory function, and gum-chewing time were measured at baseline and one month following intervention to assess outcomes.

**Results:**

One month following the intervention, tongue pressure was significantly higher in the intervention group than in the control group (*P* = 0.027). In the within-group comparisons, maximum bite force (*P* < 0.001), unstimulated saliva flow (*P* < 0.001), tongue and lip functions (/pa/: *P* < 0.001;/ta/: *P* < 0.001;/ka/: *P* < 0.001), color scale value (*P* = 0.019), and ΔE value (*P* = 0.024) were significantly increased in the intervention group.

**Conclusion:**

The results suggest that gum-chewing exercises can improve oral functions in older adults, although additional increases in masticatory load may be necessary to establish a more effective oral function training method using gum-chewing exercises in older adults.

## Introduction

The population is rapidly aging in developed countries, especially in Japan.^1^ Accordingly, the Japanese Society of Gerodontology proposed diagnostic criteria for oral hypofunction in 2016 to assess oral hypofunction in older adults.^2^ The seven diagnostic criteria for oral hypofunction are poor oral hygiene, oral dryness, decreased bite force, decreased tongue and lip functions, low tongue pressure, decreased masticatory function, and decreased swallowing function.^3^ Oral hypofunction is caused by the combined decline of several oral functions, which can be prevented by prompt diagnosis and management.^4^ Therefore, developing interventions to maintain and improve oral function in older adults is important for reducing the need for long-term care in this population.

Various intervention methods have been proposed and reported to improve oral functions of older adults.^5^, ^6^, ^7^, ^8^, ^9^, ^10^, ^11^, ^12^, ^13^, ^14^ Among these methods, chewing gum is highly palatable considering its taste,^15^ low cost, and high availability. Therefore, a gum-chewing exercise may be sustained for a long time. Furthermore, chewing gum is more hygienic because it forms a compact bolus and leaves no residue in the mouth.^16^ Because chewing gum remains elastic for a long time, it continuously loads the perioral muscles and other parts of the mouth. Thus, chewing gum may be effective as a training tool.

Several studies have investigated the effects of gum-chewing exercise on oral function in older adults.^6^^,^^14^ Nakagawa et al.^6^ reported that unstimulated saliva flow significantly increased after training intervention using soft and hard gum-chewing exercises, although bite force significantly increased only in the hard gum-chewing exercise. In a three-arm, randomized controlled trial,^14^ after an eight-week follow-up, compared to that in the control group, the change in masticatory function was significantly higher in the group that underwent oral exercise and in the group that underwent gum-chewing and oral exercise. Additionally, unstimulated saliva flow increased from baseline levels in the oral exercise and gum-chewing groups. To date, a few randomized controlled trials have investigated the effects of gum-chewing exercises on oral function in older adults.

We conducted a randomized controlled pilot study to investigate the effects of a 1-month chewing training using an author-developed chewing training gum on various outcomes related to oral function. The null hypothesis of this study was that the outcomes related to oral functions would not be significantly different between the intervention and control groups at the one-month post-intervention period and that the outcomes related to oral functions would not be significantly different between the preintervention period and one-month post-intervention period.

## Materials and methods

### Study design

This was a single-blind, randomized controlled trial, which complied with the requirements of the 2010 Consolidated Standards for Reporting Trials Statement. In addition, the study was conducted from November 2021 to January 2022 and in a single-center setting. The study protocol was approved by the Ethics Review Committee of our institution (approval number: D2020-063) and registered in the University Hospital Medical Information Network Center (UMIN-CTR Unique Trial Number: UMIN 000042470). All study participants provided written informed consent.

### Participants

The inclusion criteria were as follows: (1) age of 65–85 years, (2) complaint of difficulty in eating hard foods compared to their eating ability six months earlier, (3) independence in performing daily activities, (4) no history of secondary masticatory disturbances due to muscle diseases, (5) no problems with cognitive function, (6) no ongoing dental treatment while participating in this study, (7) at least 20 remaining teeth, (8) ability to chew gum and tablets, and (9) no gelatin allergy.

The exclusion criteria were as follows: (1) participants requested withdrawal of consent, (2) any oral problems that influenced the implementation or continuation of this study occurring during the intervention period or a participant undergoing dental treatment, (3) the intake rate of the experimental chewing gum or experimental tablet by a participant during the intervention period was <80%, or (4) the experimental chewing gum or experimental tablet intake by a participant during the intervention period was not recorded for three consecutive days.

Moreover, as a pre-intervention screening, the recruited individuals were asked to chew one piece of the experimental chewing gum twice and report the total chewing time. Based on the self-reported results, we excluded individuals who chewed the chewing gum twice for <5 min or >20 min.

### Intervention

The experimental chewing gum used in this study was stick-shaped (36 × 20 × 2.7 mm), weighed 2.0 g per piece, and was designed to allow continuous chewing at a constant hardness (2.7 ± 0.2 N), with little change in texture over time. Furthermore, the experimental chewing gum had different characteristics from the commercial gum. Firstly, the experimental chewing gum contained granules such as capsules designed to allow chewing training until all granules had been crushed. Thus, the experimental gum was chewed until the participant felt that the texture of the granules included in the gum had disappeared. Secondly, the experimental chewing gum was designed to not stick to dentures. Therefore, denture wearers could chew and perform gum-chewing training as individuals without dentures.

Participants were randomly assigned to the intervention and control groups. The intervention group was instructed to chew the experimental gum every day for one month and to chew one piece of the experimental chewing gum twice per set for a total of three sets daily. The control group was instructed to chew an experimental tablet; the group was instructed to chew two pieces of the experimental tablet twice per set for a total of three sets daily. The experimental tablet was chosen because it was necessary to use a food that could be easily adjusted its physical properties to disintegrate easily when chewed so that the training effect would not be observed in the control group.

### Outcomes

The following outcomes were assessed at baseline and one month after intervention.

#### Maximum bite force and occlusal contact areas

Maximum bite force was the primary outcome. Maximum bite force and occlusal contact areas were measured using a pressure-sensitive sheet (Dental Prescale II; GC Co., Tokyo, Japan) and an analyzer (Bite Force Analyzer; GC Co., Tokyo, Japan). Clenching was performed for 3 s at the maximal intercuspal position with a pressure-sensitive sheet placed over the occlusal surface.^17^

#### Oral dryness

Oral dryness was assessed by measuring unstimulated saliva flow. Unstimulated saliva flow is collected once in each measurement. In the measurement, saliva was spit into a disposable cup for 2 min and then weighed immediately. Considering the diurnal variation in saliva volume, saliva collection was taken at the same measurement time for each patient^18^

#### Tongue pressure

Tongue pressure was measured by a tongue pressure measuring device (TPM-02; JMS Corporation, Tokyo, Japan). Participants were instructed to press the device's balloon against their palate with maximum force for 7 s. The process was repeated three times, and the average value was analyzed.^19^^,^^20^

#### Tongue and lip functions

The number of times a syllable (i.e.,/pa/,/ta/, and/ka/) was pronounced per second was measured using an automatic measuring device (Kenko-kun Handy, Takei Kiki Kogyo Co., Niigata, Japan).^21^

#### Masticatory function

Objective assessment of masticatory function was conducted by color-changing chewing gum (Masticatory Performance Evaluating Gum XYLITOL; Lotte Co., Tokyo, Japan). Measurements were performed using a 10-point color scale using a colorimeter (CR-20; KONICA MINOLTA, Tokyo, Japan), and the respective measurements (i.e., color scale value and ΔE value) were obtained.^22^^,^^23^ Moreover, subjective assessment of masticatory function was performed by obtaining the mastication score using a food intake questionnaire.^24^^,^^25^

#### Gum-chewing time

Gum-chewing time was measured to investigate the actual time spent by participants to chew the gum. The time required for a participant to finish chewing an experimental chewing gum twice was measured. Participants were asked to self-report when the granules in the gum had disappeared.

### Sample size

The sample size was calculated based on a previous study.^10^ The difference in maximum bite force before and after training in the intervention group was set at 110.0 N, and the standard deviation was 207.2 N. Furthermore, the significance level, power, and effect size were set to 5%, 80%, and 0.5, respectively. Consequently, the sample size was determined to be 114 participants. Thus, considering an exclusion rate of 20% and a dropout rate of approximately 20%, 180 participants were required for this study.

### Randomization and blinding

In this single-blind study, the assessors were blinded. The study participants were randomly assigned using a stratified block method with sex and age as stratification factors by individuals who were not the assessors.

### Statistical analysis

Between-group comparisons were performed using the chi-square test for sex and Mann–Whitney *U* test for other data. Within-group comparisons were performed using the Wilcoxon signed-rank test. All statistical analyses were performed using SPSS (version 23.0; IBM Corp., Armonk, NY, USA). *P* < 0.05 was considered statistically significant.

## Results

### Participants

**Figure 1:**
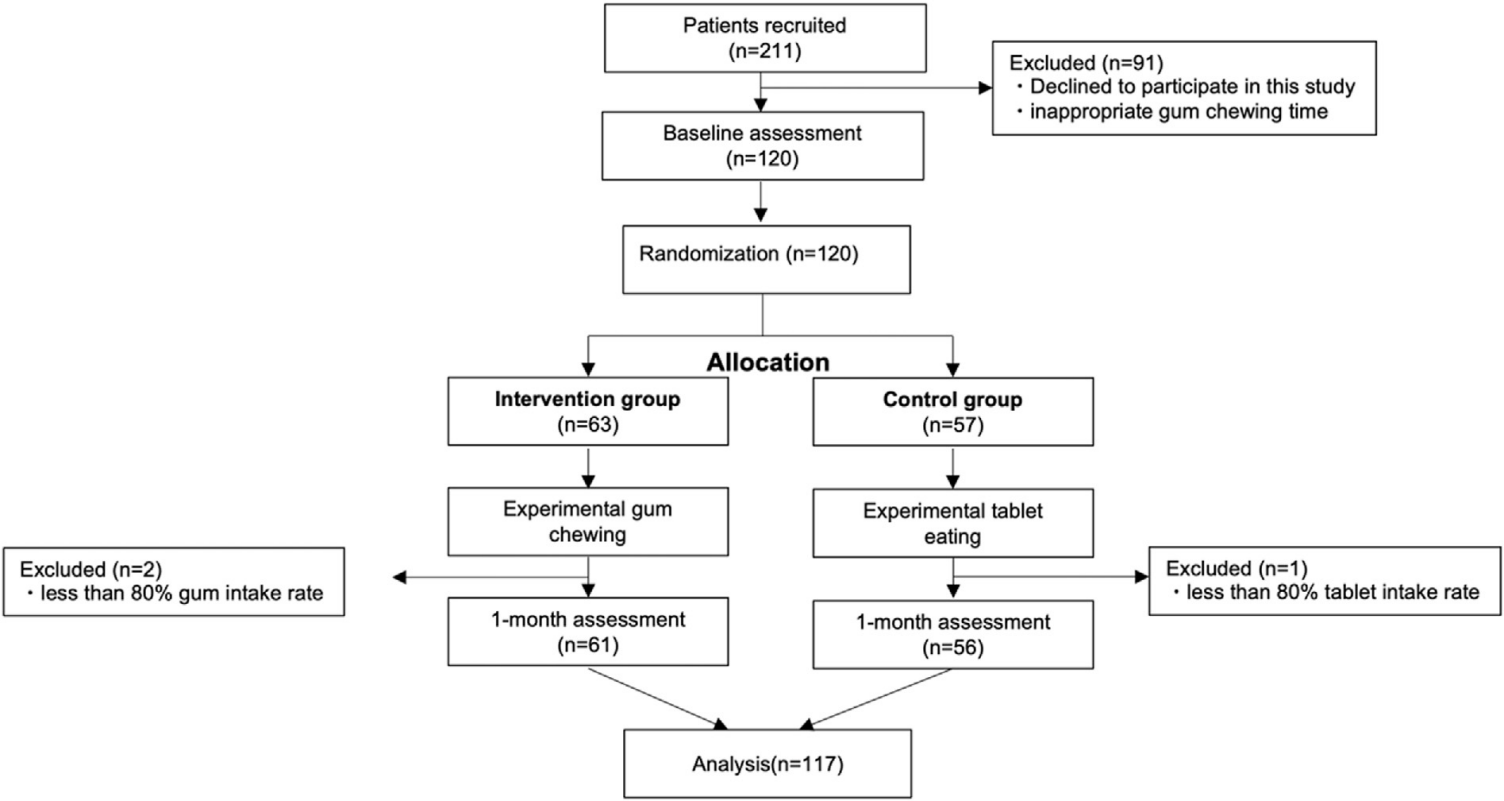
Consolidated Standards of Reporting Trials (CONSORT) flow chart of study participants.

Fig. 1 shows the flow diagram of the inclusion and exclusion of study participants. Baseline characteristics of the participants are shown in Table 1. No significant differences existed in the baseline characteristics between the 61 and 56 participants in the intervention and control groups, respectively.

**Table 1:** Baseline characteristics of study participants.

	Intervention (n = 61)	Control (n = 56)	*P*-value
Age, years^a^	70.8 (4.5)	70.8 (3.9)	0.718^b^
Sex (%)			0.995^c^
Female	24 (39%)	22 (39%)	
Male	37 (61%)	34 (61%)	
Body height, cm^a^	163.7 (9.3)	163.9 (9.7)	0.996^b^
Body weight, kg^a^	63.1 (11.9)	62.0 (12.3)	0.676^b^
Number of teeth^a^	26.9 (2.1)	26.7 (2.6)	0.987^b^
Number of functional teeth^a^	27.8 (1.5)	27.7 (1.9)	0.953^b^
Number of denture wearers	7 (11%)	7 (13%)	0.864^c^

a Data are presented as mean (standard deviation).

b Based on the *t*-test.

c Based on the chi-square test.

### Measurement of oral function

Pre- and post-intervention outcomes are summarized in Table 2 for between-group comparison and in Table 3 for the within-group comparison.

**Table 2:** Between-group differences in the median (interquartile range) baseline and 1-month post-intervention results of oral functions.

	Baseline			1 M		
	Intervention (n = 61)	Control (n = 56)	*P*-value	Intervention (n = 61)	Control (n = 56)	*P*-value
Maximum bite force (N)	548.2 (474.0)	575.8 (414.9)	0.297	641.7 (503.5)	609.1 (496.5)	0.372
Occlusal contact areas (mm^2^)	15.1 (12.4)	14.3 (10.4)	0.206	16.9 (13.6)	15.3 (12.0)	0.364
Unstimulated saliva flow (g)	1.9 (1.3)	2.0 (1.3)	0.961	2.2 (1.0)	2.2 (1.2)	0.530
Tongue pressure (kPa)	31.9 (9.4)	29.3 (8.0)	0.080	32.3 (8.0)	30.0 (6.0)	0.027∗
Tongue and lip functions						
/pa/(times)	6.2 (0.8)	6.0 (1.0)	0.440	6.3 (0.8)	6.4 (1.0)	0.699
/ta/(times)	6.0 (1.0)	6.2 (1.4)	0.967	6.2 (0.8)	6.4 (1.4)	0.749
/ka/(times)	5.8 (1.0)	5.8 (1.0)	0.734	6.0 (0.8)	6.0 (1.2)	0.742
Color-changing chewing gum						
Color scale	9.0 (1.0)	9.0 (1.0)	0.236	10.0 (1.0)	9.0 (1.0)	0.056
ΔE	48.8 (5.9)	47.9 (4.6)	0.974	49.4 (5.7)	47.7 (6.1)	0.179
Mastication score	90.3 (13.3)	93.5 (14.3)	0.305	93.5 (11.7)	93.5 (13.3)	0.479
Masticatory time (mins)	2.5 (1.3)	2.3 (1.3)	0.426	2.1 (0.6)	2.0 (1.1)	0.956

1 M, 1-month assessment. Data are presented as medians (interquartile range).

∗ Significant difference (*P* < 0.05).

**Table 3:** Within-group differences in the median (interquartile range) baseline and 1-month post-intervention results of oral functions.

	Intervention (n = 61)			Control (n = 56)		
	Baseline	1 M	*P*-value	Baseline	1 M	*P*-value
Maximum bite force (N)	548.2 (474.0)	641.7 (503.5)	<0.001∗	575.8 (414.9)	609.1 (496.5)	<0.001∗
Occlusal contact areas (mm^2^)	15.1 (12.4)	16.9 (13.6)	0.052	14.3 (10.4)	15.3 (12.0)	0.024∗
Unstimulated saliva flow (g)	1.9 (1.3)	2.2 (1.0)	<0.001∗	2.0 (1.3)	2.2 (1.2)	<0.001∗
Tongue pressure (kPa)	31.9 (9.4)	32.3 (8.0)	0.167	29.3 (8.0)	30.0 (6.0)	0.244
Tongue and lip functions						
/pa/(times)	6.2 (0.8)	6.3 (0.8)	<0.001∗	6.0 (1.0)	6.4 (1.0)	<0.001∗
/ta/(times)	6.0 (1.0)	6.2 (0.8)	<0.001∗	6.2 (1.4)	6.4 (1.4)	<0.001∗
/ka/(times)	5.8 (1.0)	6.0 (0.8)	<0.001∗	5.8 (1.0)	6.0 (1.2)	0.047∗
Color-changing chewing gum						
Color scale	9.0 (1.0)	10.0 (1.0)	0.019∗	9.0 (1.0)	9.0 (1.0)	0.132
ΔE	48.8 (5.9)	49.4 (5.7)	0.024∗	47.9 (4.6)	47.7 (6.1)	0.980
Mastication score	90.3 (13.3)	93.5 (11.7)	0.078	93.5 (14.3)	93.5 (13.3)	0.820
Masticatory time (mins)	2.5 (1.3)	2.1 (0.6)	<0.001∗	2.3 (1.3)	2.0 (1.1)	<0.001

1 M, 1-month assessment. Data are presented as median (interquartile range).

∗ Significant difference (*P* < 0.05).

In between-group comparisons at pre-intervention, there were no significant differences between the intervention and control groups in any outcomes.

In between-group comparisons at post-intervention, the intervention group had significantly higher tongue pressure than the control group (*P* = 0.027).

Within-group comparisons of the intervention group showed significant increases in maximum bite force (*P* < 0.001), unstimulated saliva flow (*P* < 0.001), tongue and lip function (/pa/: *P* < 0.001;/ta/: *P* < 0.001;/ka/: *P* < 0.001), color scale values (*P* = 0.019), ΔE values (*P* = 0.024), and gum chewing time (*P* < 0.001).

Within-group comparisons of the control group showed significant increases in maximum bite force (*P* < 0.001), unstimulated saliva flow (*P* < 0.001), tongue and lip function (/pa/: *P* < 0.001;/ta/: *P* < 0.001;/ka/: *P* = 0.047), and gum chewing time (*P* < 0.001).

## Discussion

The null hypothesis was rejected for tongue pressure in the between-group comparison one month after intervention. In the within-group comparisons of the intervention group, the null hypothesis was rejected for maximum bite force, unstimulated salivary flow, tongue and lip functions (/pa/,/ta/,/ka/), color scale value, and ΔE value.

Herein, a comprehensive evaluation of various outcomes related to oral function, including hypofunction diagnostic items, was conducted to investigate the effects of chewing training using a gum that was developed to prevent and improve hypofunction of the oral cavity. Furthermore, the training method in this study does not require chewing for a certain period of time or self-counting the number of times chewed; thus, it is considered simple and easy-to-maintain for older adults. Notably, only two participants were excluded from the study for having <80% adherence to the regimen.

The primary outcome of this study, maximum bite force, did not differ significantly between groups. Participants who chewed a piece of the gum twice in <5 min were excluded before intervention. However, the median chewing time in the intervention group was 6–7 min daily. A prospective cohort study,^10^ which examined the effects of one-month gum-chewing exercise twice daily, with each set comprising 5 min of chewing, in adults, revealed a significant increase in maximum bite force. A randomized controlled study^13^ showed a significant increase in maximum bite force after one-month gum-chewing exercises thrice daily, with each set comprising 5 min of chewing, in junior high school students. Therefore, the participants in this study compared to those in the previous study, spent less chewing time during training, which may be the reason for the lack of a significant difference in maximum bite force in this study. Conversely, in a randomized controlled trial investigating the effects of gum-chewing exercise in older adults, Kim et al.^14^ found no significant difference in the change in bite force between the gum-chewing and control groups, as in our study. In their study, participants underwent gum-chewing exercise twice daily for 10 min each time, thereby training for 20 min daily. Compared to youths and adults, older adults possibly need more chewing time during gum-chewing exercise to increase bite force. However, the effective and appropriate chewing time that enables older adults to continue gum-chewing exercises should be examined.

In the between-group comparison, the intervention group had significantly higher tongue pressure than the control group after one month of intervention. However, within-group comparisons of the intervention and control groups revealed no significant differences. The median tongue pressures of the intervention group at pre- and post-intervention showed a change of only 1.3%. Furthermore, the mean age of the intervention group in this study was 70.8 years, while the mean tongue pressure in previous studies was reportedly 31.9 ± 8.9 kPa for individuals in their 70s.^19^ Therefore, it is not possible to state that the significant difference in tongue pressure between groups in this study was a clinically significant value. However, tongue pressure is related to masticatory performance^26^ and swallowing^27^; therefore, an increase in tongue pressure by a gum-chewing exercise may contribute greatly to maintaining and improving oral hypofunction.

Within-group comparisons showed significant increases in several outcomes at pre- and post-intervention. The only outcomes that increased significantly in the intervention group were the color scale and ΔE value, which are indices to assess masticatory function. Several factors related to masticatory function, including occlusal contact area,^28^ maximum bite force,^29^, ^30^, ^31^, ^32^ tongue and lip functions,^33^ mandibular movements,^34^, ^35^, ^36^, ^37^ and muscle activity of masticatory muscles,^38^ have been reported. However, in this study, maximum bite force and tongue and lip functions increased significantly in both groups, making it difficult to identify factors that contributed to the increase in color scale and ΔE values only in the intervention group. A previous study has indicated that masticatory function with color-changing chewing gum may be affected by habituation to gum chewing itself.^15^ Therefore, habituation to gum chewing may be the reason for the significant increase in color scale and ΔE values in the intervention group in this study.

This study had several limitations. First, the control group underwent a dummy intervention in which tablets were continuously consumed. Contrary to our expectations, several outcomes were significantly increased in the control group one month after intervention. Several reasons exist for this finding. Firstly, the Hawthorne effect on the participants may have influenced the increase. The Hawthorne effect is the statistical appearance of an improvement in symptoms when patients consciously or unconsciously fail to report their symptoms or behave in a way that suggests that their symptoms have improved, in order to meet the expectations of a trusted doctor or other health professional.^39^ Secondly, the experimental tablets used in this study were easily disintegrated by chewing but had a certain degree of hardness, which may have mimicked the effect of the gum-chewing exercise. This suggests that continuously chewing foods with a certain degree of hardness, including the tablets used herein, is effective in maintaining and improving oral functions. Second, the final sample size of this study was 117 participants. A post-hoc test showed that the effect size of this study was 0.16, which was inadequate when the power was set at 0.8 at a 5% significance level. Therefore, the sample size was small. However, the effect size can be increased by improving the experimental gum and changing the gum-chewing method, in addition to increasing the sample size. Third, because the method depended on the participants' subjectivity, the average chewing time of the training gum of the participants in the intervention group was shorter than the expected chewing time, which might cause a decrease in the training effect of gum chewing in the intervention group and show no significant differences in most of the outcomes including maximum bite force, between the groups. In the future, it is necessary to consider a way for individuals to confirm that they have chewed until the granules are properly removed.

In conclusion, under the limited conditions of this study, the results suggest that gum-chewing exercises can improve oral functions in older adults, although additional increases in masticatory load may be necessary to establish a more effective oral function training method using gum-chewing exercises in older adults. Therefore, this pilot study could serve as a useful indicator for the development of oral function training methods using gum-chewing exercises.

## Declaration of competing interest

The authors have no conflicts of interest relevant to this article.
